# Brief Narrative Review on Commercial Dental Sealants—Comparison with Respect to Their Composition and Potential Modifications

**DOI:** 10.3390/ma16196453

**Published:** 2023-09-28

**Authors:** Aleksandra Piszko, Paweł J. Piszko, Adam Lubojański, Wojciech Grzebieluch, Maria Szymonowicz, Maciej Dobrzyński

**Affiliations:** 1 Department of Pediatric Dentistry and Preclinical Dentistry, Wroclaw Medical University, Krakowska 26, 50-425 Wrocław, Poland; adam.lubojanski@student.umw.edu.pl (A.L.); maciej.dobrzynski@umw.edu.pl (M.D.); 2 Department of Polymer Engineering and Technology, Faculty of Chemistry, Wrocław University of Science and Technology (WUST), wyb. Wyspiańskiego 27, 50-370 Wrocław, Poland; 3 Laboratory for Digital Dentistry, Department of Conservative Dentistry with Endodontics, Wroclaw Medical University, Krakowska 26, 50-425 Wroclaw, Poland; 4 Pre-Clinical Research Centre, Wroclaw Medical University, wyb. Ludwika Pasteura 1, 50-367 Wrocław, Poland; maria.szymonowicz@umw.edu.pl

**Keywords:** sealants, dental, fluoride, marginal integrity, caries prevention, resin-based sealants, composition

## Abstract

The scope of this paper is to compare different dental sealants and flow materials indicated for sealing pits and fissures considering their chemical formula. The narrative review aims to address the following questions: What is the essence of different dental sealants’ activity, how does their chemical formula affect their mechanisms of caries prevention, and what makes a dental sealant efficient mean of caries prevention? Another vital issue is whether the sealants that contain fluoride, or any other additions, have potentially increased antimicrobial properties. An electronic search of the PubMed, Cochrane, Web of Science, and Scopus databases was performed. The following keywords were used: (dental sealants) AND (chemical composition). Additionally, information about composition and indications for clinical use provided by manufacturers were utilized. All of the considered materials are indicated for use both in permanent and primary dentition for sealing fissures, pits, and *foramina caeca*. The selection of suitable material should be made individually and adjusted to conditions of the sealing procedure and patient’s needs. Cariostatic mechanisms increasing sealants’ effectiveness such as fluoride release are desired in modern dentistry appreciating preventive approach. The review aims are to find crucial elements of sealants’ composition which affect their cariostatic mechanisms.

## 1. Introduction

The World Health Organization (WHO) states that dental caries is a major public health problem worldwide and is the most widespread non-communicable disease (NCD). Moreover, the WHO states that “Dental caries can be prevented by avoiding dietary free sugars (…) is largely preventable through simple and cost-effective population-wide and individual interventions, whereas treatment is costly, and is often unavailable in low- and middle-income countries” [1]. A systematic review on the global burden of untreated caries can be referenced to present the scale of untreated caries phenomena. It reported that between 1990 and 2010, caries prevalence worldwide affected 2.4 billion people [2]. Over recent years, methods of prevention of tooth decay are shifting towards minimal intervention dentistry. Tendency to preserve as many natural tooth tissues as possible as well nonrestorative approach are promoted [3].

**Figure 1:**
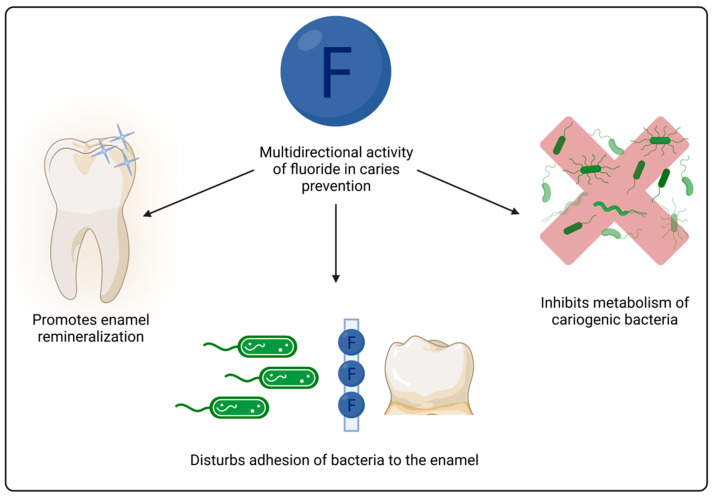
Graphical depiction of fluoride activity in caries prevention.

Fluoride is an important factor in caries prevention. It has a proved impact both on cariogenic bacteria and on maintaining balance between the processes of demineralization and remineralization. It may disturb bacteria’s metabolism and adherence to the enamel. Moreover, fluoride ions, when present in saliva in a sufficient amount, delay demineralization and promote enamel remineralization [3]. The mechanism of enamel remineralization consists of replacing the -OH groups of hydroxyapatite with fluorine [4]. Additional, thanks the supply of fluoride, calcium fluoride and fluoridated carbonato-apatite are also formed [5]. Due to the presence of fluoride in human saliva, the likelihood of tissue remineralization increases. Developed biofilm blocks the access of fluoride inside the tooth tissues [6]. Therefore, in case of more effective fluoride agents, it should be removed. Finally, the process of remineralization is facilitated with increased porosity of the tissue affected by caries. It is worth mentioning that antibacterial properties of fluoride are as important as the process of remineralization of hard tooth tissues [7]. The mechanism of fluoride’s antibacterial action considers diffusion of fluoride ions into the bacterial cell. At acidic pH values, enolase and adenosine triphosphatase enzymes are inactivated. Fluoride effectively inhibits the carbohydrate metabolism of acidogenic oral bacteria which also includes the uptake of sugars [8]. The aforementioned multidirectional activity of fluoride is depicted graphically on Figure 1.

A role of saliva should be mentioned while discussing caries prevention. Saliva has an ability to eliminate sugars and other substances, buffer capacity, balance demineralization/remineralization, and antimicrobial action [9]. Cariogenic bacteria levels within the saliva and plaque determine whether caries will occur or not [10]. In some specific conditions like xerostomia, which is a complaint of oral dryness, the risk of oral infection may be superior [11,12]. The prevalence of xerostomia described also as a hypo-salivation” or “dry mouth” is 14 to 46% and more frequently considers women [13,14]. It is a particular condition in which even more attention should be paid to managing plaque retention and caries prevention.

Dental sealants were first introduced in the 1960s, in scope of helping to prevent dental caries, mainly in the pits and fissures of occlusal tooth surfaces [15]. Since their introduction to the market, sealants are frequently mentioned as dental materials serving for caries prevention and managing early caries lesions [3]. Their effectiveness in preventing and detaining pit-and-fissure occlusal carious lesions of primary and permanent molars was concluded in a clinical guideline by the American Dental Association and the American Academy of Paediatric Dentistry [16]. Materials dedicated for sealing are indicated by the manufacturers to use both in permanent and primary dentition for covering fissures, pits, and *foramina caeca*. Contradictions are allergies or hypersensitivity for any ingredient of the material. The inability to keep the operative field dry may be a contradiction for some materials, mainly resin-based sealants and flow composites, while it is not an issue while using other materials, such as glass-ionomers. The material should be chosen according to the patient’s needs, operator preferences, and best and current medical knowledge. The aim of this narrative review is to summarize the information on fissure sealants with particular emphasis on their composition as well as physicochemical and biological properties. Another important aspect covered by this paper is the effectiveness of materials used for sealing and factors influencing their durability.

## 2. Methods 

The review revolves around the following questions: What is the essence of action of different dental sealants and how does their composition affect their effectiveness in caries prevention? Do the sealants contain fluoride and by means of what mechanisms do they release it to the environment of the oral cavity? What factors influence the durability of materials for sealing? An electronic search of the PubMed, Cochrane, Web of Science, Google Scholar, and Scopus databases was performed. The following keywords were used: (dental sealants) AND (chemical composition) according to MeSH terms. Papers considering surface sealants, coating, or other materials where dental sealants were just mentioned were excluded from the review. Information about chemical composition of some commonly used dental sealants provided by manufactures were found.

## 3. Results

### 3.1. Comparison of Composition of Commercially Used Materials

Commercial sealants differ in the composition of matrix, added fillers, or presence of fluoride. Their structure directly influences their properties. Therefore, a literature-based comparison of 19 different commercial sealants was performed. Juxtaposition was executed with respect to product name, abbreviation (for the sake of discussion), manufacturer, composition, and presence of fluoride (Table 1). Furthermore, the experimental properties influencing their clinical performance included shear bond strength, hardness, and shrinkage. The selection of the materials presented in Table 1 was based on the availability of the sealants’ characteristics in the literature and manufacturers data. The references include peer-reviewed publications as well as Material Safety Data Sheets of the materials and other data shared by manufacturers. The table is lacking in parameters for few materials which indicates the gap in knowledge for further research on commercial materials. Furthermore, one must bear in mind that availability of experimental data on exact dental sealants is very limited. Therefore, the juxtaposition of parameters in Table 1 does not include differences in experimental methodologies.

The shear bond strength of the analyzed sealants ranged from 3.5 ± 0.8 MPa for FT up to 42.6 ± 3.2 for GS. In terms of hardness, HF possesses the lowest value (19.3 HV) and EGF the highest (99.3 ± 4.5 HV). General observation related higher HV values with the presence of polyacrylic acid as one of the main ingredients in the sealant composition (e.g., EQF, IMAC, KM).

Ultimately, shrinkage of the presented materials is within the range of 1.95–7.40%, the lowest end being TEC and the highest TF1. 

The composition of resin-based materials influences their properties. Modifications to the matrix contribute to a shrinkage change depending on the main polymers used in the matrix. Conventional resin-based materials have polymerization shrinkage caused by the approximation of monomers during polymerization. However, volumetric changes of restorations also depend on the stress generated at the tooth–restoration interface while the material is undergoing shrinkage As mentioned in an example of literature, both Bis-GMA, UDMA, and TEGDMA usually undergo moderate to severe polymerization shrinkage due to their free radical polymerization reaction [17,18]. The highest shrinkage values were obtained for materials containing TEGDMA as a main or one of the main polymer constituents of the sealant (7.40% for TF1, 6.60% for CS, 5.98% for USXT). Only for FFX containing TEGDMA was the shrinkage value slightly lower (4.30%), probably due to the higher concentration of Bis-GMA (up to 10%) which not present in other specimens at all or in such high amounts.

**Table 1:** Juxtaposition of commercially used materials dedicated for sealing.

Product Name	Abbr.	Manufacturer	Composition	Fluoride Presence	Shear Bond Strength [MPa]	Hardness [HK] or [HV]	Shrinkage [%]	Light Curing	Material Classification	Ref.
Helioseal F	HF	Ivoclar Vivadent, Lichtenstein	bisphenol A-glycidyl methacrylate (Bis-GMA), dimethacrylates fluorosilicate glass, silica, titanium dioxide, initiators, and stabilizers	Yes	13.7 ± 7.0	19.3 HV	3.98	Yes	Sealant	[19,20]
Helioseal	HS	Ivoclar Vivadent, Lichtenstein	Bis-GMA, triethylene glycol dimethacrylate (TEGDMA), titanium dioxide, stabilizers, and catalysts	No	12 ± 1.0	-	-	Yes	Sealant	[21,22]
Fissure Sealant	FS	Arkona	Bis-GMA, TEGDMA, Urethane dimethacrylate (UDMA), barium- aluminum-silicon glass, barium- aluminum-boron-fluorine glass, fire silica, photoinitiators (CQ:DMAEMA-camphorquinone:ethyl-4-dimethylaminobenzoate), stabilizers, pigments	Yes	-	-	-	Yes	Sealant	[23]
Embrace Wetbond	EW	Pulpdent, United States	Uncured acrylate ester monomers 55–60%, amorphous silica 5%, sodium fluoride < 2%	Yes	21.7 ± 2.0	23.9 HV	3.45	Yes	Sealant	[19,24,25]
Fuji Triage	FT	GC Cooperation, Japan	Glass-ionomer, aluminofluorosilicate glass, polyacrylic acid, distilled water, polybase carboxylic acid	Yes	3.5 ± 0.8	52.0 ± 1.0 HV *	-	Yes/No ***	Glass-ionomer	[26,27]
Smart Seal loc F	SSLF	Detax, Germany	bis(methacryloxyethyl) hydrogen phosphate, 2-propenoic acid, 2-methyl-2-hydroxyethyl ester, phosphate, 2-dimethylaminoethyl methacrylate	Yes	9.5 ± 1.4	-	5.06 ± 1.20	Yes	Sealant	[28,29]
Fuji VII EP	F7E	GC Cooperation, Japan	Fluoroaluminosilicate glass, casein phosphopeptide-amorphous calcium phosphate (CPP-ACP), pigment, distilled water, polyacrylic acid, polybase carboxylic acid	Yes	5.0 ± 1.7	47.1 ± 6.0 HV	-	No	Glass-ionomer cement	[26,30]
GCP Glass Seal	GCP	GCP Dental, Netherlands	Nanoparticles glass ionomer-based material	Yes	-	50.0 ± 1.5 HV	-	Yes/Nos	Glass-ionomer sealant	[27,31]
Ketac Molar	KM	3M ESPE, Germany	Al-Ca-La fluorosilicate glass, 5%, copolymer of acrylic acid and maleic acid, polyacrylic acid, tartaric acid, water	Yes	4.8 ± 1.0	89.9 ± 4.2 HV	-	No	Glass-ionomer	[32,33]
Voco Ionofil Molar AC Quick	IMAC	Voco, Germany	Water, polyacrylic acid, (+)-tartaric acid, aluminofluorosilicate glass, and pigments	Yes	5.3 ± 0.6	79.9 ± 2.1 HV	-	No	Glass-ionomer	[32,34,35]
Equia Fil	EQF	GC Cooperation, Japan	Polyacrylic acid, aluminosilicate glass, distilled water	No	-	99.3 ± 4.5 HV	-	No	Glass-ionomer	[32]
UltraSeal XT plus	USXT	Ultradent, USA	TEGDMA 10–25%, diurethane dimethacrylate 2.5–10%, aluminium oxide 2.5–10%, 2-hydroxyethyl methacrylate < 2.5%, amine methacrylate < 2.5%, organophosphine oxide < 2.5%, sodium monofluorophosphate < 0.1%	Yes	42.7	27.6 HK	5.98	Yes	Sealant	[36,37]
Conseal F	CF	SDI, Australia	UDMA base 7% filled with a submicron filler size of 0.04 µm	No	14.0 ± 0.9	-	-	Yes	Sealant	[28]
Tetric Flow	TF	Vivadent	Bis-GMA (10–25%), UDMA (10–25%), ytterbium trifluoride, 1,10-decandiol dimethacrylate (2.5–10%), diphenyl(2,4,6- trimethylbenzoyl)phosphine oxide (0.1–2.5%), 2-(2-Hydroxy-5-methylphenyl)-benzotriazol; 2-(2*H*-Benzotriazol-2-yl)-p-kresol (0.1–1.0%)	Yes	16.8 ± 2.7	34.0 HV **	-	Yes	Flow composite	[20,38,39]
Tetric Evo Ceram	TEC	Vivadent	Dimethacrylate co-monomers (17–18 wt.%), barium glass, ytterbium trifluoride, mixed oxides and prepolymers (82–83 wt.%)	Yes	20.7 ± 7.2	51.0 HV **	1.95 ± 0.03	Yes	composite	[38,40,41]
Wave	WV	SDI, Australia	UDMA, strontium glass	No	24.6 ± 1.5	-	5.00	Yes	Flow composite	[42,43]
Clinpro Sealant	CS	3M ESPE, Germany	TEGDMA, bisphenol A digilycidyl ether dimethacrylate, tetrabuttylammonium tetrafluoroborate, silane-treated silica	Yes	12.8 ± 8.3	21.5 ± 0.2 HV	6.60 ± 1.54	Yes	Sealant	[29,44,45]
Grandio Seal	GS	Voco, Germany	TEGDMA (10–25%), fumed silica (5–10%), Bis-GMA (2.5–5%)	No	42.6 ± 3.2	75.1 ± 2.0 HV	-	Yes	Sealant	[45,46,47]
Fissurit FX	FFX	Voco, Germany	TEGDMA (15–25%), Bis-GMA (5–10%), sodium fluoride (≤2.5%)	Yes	6.2 ± 0.7	-	4.30 ± 1.15	Yes	Sealant	[29,48,49]
Dyract Seal	DS	Dentsply, Germany	Patented macromonomers (AP and M-1A-BSA), strontium-aluminium, fluorosilicate glass, Diethylene glycol dimethacrylate DGDMA, dispersed silicon oxide (Aerosil), initiators, inhibitor	Yes	8.3 ± 0.3	-	5.38 ± 1.30	Yes	Sealant	[29,50]
Teethmate F-1	TF1	Kuraray, Japan	2-hydroxyethyl methacrylate, TEGDMA, 10-methacryloyloxydecyl dihydrogen phosphate, methacryloylfluoride-methyl methacrylate copolymer, hydrophobic aromatic dimethacrylate, d,l-camphorquinone, initiators, accelerators, dyes	Yes	-	26.7 ± 1.3 HV *	7.40 ± 1.17			[27,29]

* Recalculated from GPa. ** Converted from HK. *** White version—chemical polymerization, pink version—light polymerization.

### 3.2. Modifications in Composition

Biomaterials used in dentistry are constantly evaluated to meet the demands of clinical needs. Non-invasive approaches and prevention are promoted in modern dentistry. Modifications of materials are performed to enhance their clinical behavior. Among the desired traits of materials dedicated for sealing, we may list durability, ease of application, or bacteriostatic potential.

In order to decrease biofilm viability on the surface of resin-based sealant, its composition can be altered. A study presents that doping a methacrylate monomer matrix with 2.5 wt.% of 1,3,5-triacryloylhexahydro-1,3,5-triazine (TAT) in dental sealant’s structure impacts its cytotoxicity, biofilm formation, and physicochemical properties [51]

Another example of structural modification of dental sealant available on the market includes incorporation of methacryloxylethyl cetyl dimethyl ammonium chloride (DMAE-CB) into Helioseal pit and fissure sealant [52]

The antibacterial action of a dental material may be also obtained by an incorporation of acrylated hydroxyazobenzene (AHA) copolymers into a composite-resin matrix [53]. The study was based on samples of bisphenol A-glycidyl methacrylate and triethylene glycol dimethacrylate (bisGMA:TEGDMA) with and without AHA doping. It resulted in the same level of biocompatibility of both materials. Moreover, an inhibitory effect of AHA addiction on *Streptococcus mutans* biofilm growth was observed [54]. The authors claim that AHA may be incorporated into restorative and sealing materials in order to increase anticaries potential of dental materials.

**Figure 2:**
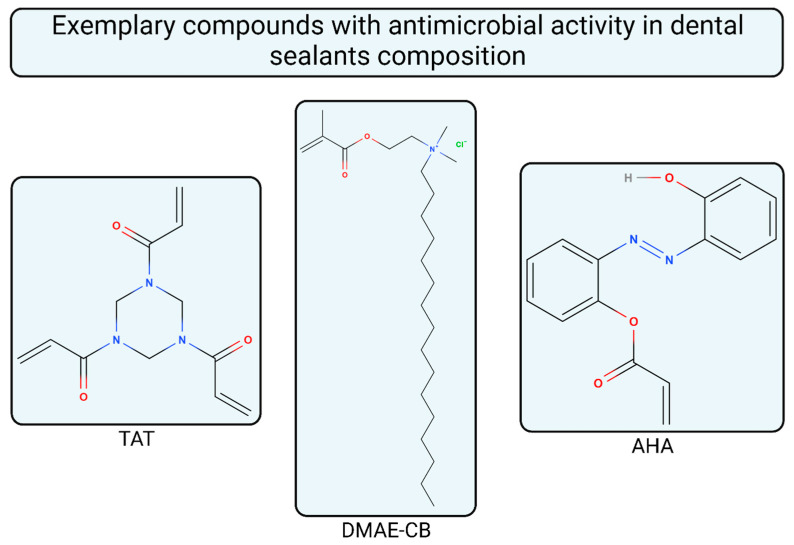
Exemplary chemical structure of compounds with antimicrobial properties used in dental sealants including TAT, DMAE-CB, and AHA.

As we may see in the Table 1, some of the materials for sealing contain fluoride in different forms, whereas others do not have it in their composition at all. The role of fluoride ions in caries prevention is important and was briefly described in the introduction paragraph. The study comparing sealant containing fluoride and without it showed differences in enamel hardness after cariogenic challenge [55]. Experiment was performed on blocks of human third molars and showed no significant differences between materials in the aspect of marginal adaptation. However, it concluded that using fluoride sealant is recommended to prevent caries in high-caries-risk patients because of its favorable impact on enamel’s hardness decrease. The chemical structure of aforementioned compounds used for doping sealing materials is depicted in Figure 2.

### 3.3. Indications for Use

All of the considered materials defined as dental sealants are described by manufacturers as indicated to seal fissures, pits, and *foramina caeca* of primary and permanent teeth. Some of the materials are also recommended for managing early caries lesions. At the same time, allergies or hypersensitivity for any ingredient of the material are defined as contraindications. Resin-based materials for sealing are not indicated in case of inability to keep the operative field dry (e.g., HF, WV). In that case, glass-ionomer materials are applied for sealing (e.g., KM). Moreover, while using resin-based materials, direct contact with preparations containing phenolic compounds, especially eugenol or thymol should be avoided. These compounds may disturb the polymerization [23]. At the same time, resin-based materials polymerize “on demand” during light curing and are more resistant. Flow composite indicated as suitable for sealing or releasing fluoride were also taken into consideration in this study. Some of the materials are clearly indicated to extended fissure sealing, e.g., TF. Among the analyzed dental sealants that contain fluoride are HF, EW, FT, SSLF, F7E, GCP, KM, IMAC, USXT, TF, TEC, CS, FFX, DS, and TF1. Each material is presented by its manufacturer as superior and suitable for many conditions in patients’ oral cavity. There are certain characteristics which distinguish materials among each other and make them more suitable for certain clinical applications. The materials have different colors and translucency which determines their appearance in the oral cavity. Colorful materials may be more attractive for children and increase their involvement into dental treatment. Moreover, their retention is easier to control by the parents. On the other hand, colorless (e.g., TF1) materials can be perceived as more aesthetic, especially among adult patients. Another highly aesthetic solution is the application of a flow composite that has different color shade variants, such as WV. Most manufacturers of materials for sealing contain in the safety data sheet a warning for users against inhalation, skin contact, eye contact, or swallowing the material.

The review, based on several examples of research, concluded that caries may be avoided in 60% of sealed surfaces [56]. At the same time, the author notices that the beneficial effect is more significant in populations with high caries baseline risk. The article points out that both resin and glass ionomer sealants are indicated as effective methods of caries prevention. The time that is indicated as the most appropriate for sealing is the first year after eruption of the first molar and the first two to three years regarding the second molar. 

Dental sealants are frequently described as materials indicated to prevent caries and manage early carious lesions [3]. Once again, it is concluded that the most beneficial effect of sealing is obtained in groups, mostly children, characterized by a high caries risk. Among the limitations of optimal sealant application, the authors list operators’ and cooperations’ dependent factors, such as optimum isolation, cleaning of the tooth surface, and etching.

### 3.4. Microleakage and Adhesion

One of the desired features of restorative material is its durability. It depends on many factors and adhesion is one of them. The criteria considered in a number of studies is marginal adaptation and microleakage as a factor describing the material’s potential of durability and efficiency. Adhesion depends not only on the material itself but also on widely recognized surface preparation. Conditioning of the surface should provoke better retention of the material, which is mainly mechanical as the physico-chemical interaction between the resin and etched enamel is small [57]. The essence of its action considering hard dental tissues is based on filling the pores by resin monomers that are polymerized and are interlocked [58]. Etching has an impact on enamel dissolving rods and creating microporosities which can be penetrated by a material [59]. Adhesion, which enables junction between dental material and hard tissues of the tooth, is also a part of the aforementioned non-invasive approach as it promotes preserving sound dental structures. Producers usually provide users of the sealer with instructions containing indications for use. Most of them advise to etch the enamel surface prior to applying a material with 37% phosphoric acid for around 30 s (e.g., for 20–40 s such as in the case of Arkona dental sealant) [23]. It is a protocol commonly followed by clinicians that has support in numerous studies and sheets for the users attached to materials for sealing [60,61]. It is worth noticing that shrinkage of sealants is generally lower with the increasing amount of filler in the material. The chance of filling leaks also drops [62]. In addition, Rahimian-Imam S et al. mentions that self-adhering sealants have lower leakage than conventional materials. This is particularly useful in clinical applications because it can eliminate the problem of maintaining proper conditions even with uncooperative patients [63].

Many studies aim to find the best way of conditioning the enamel surface so that pit and fissure sealing is most effective, i.e., has the lowest microleakage or better retention. The comparison of acid-etching, laser, or a combination of them both was tested in the contest of different sealants [64,65,66,67]. All the above-mentioned studies concern human teeth and natural enamel tissue. Most of them were performed on extracted teeth, apart from one, which was conducted in the oral cavity of patients and controlled for one year [66]. This study concluded that both ways of conditioning—laser and acid-etching—are successful in promoting sealant retention, and in all cases there were no secondary caries detected. The studies on extracted teeth used artificial sample ageing by using thermocycling and a water bath. The microleakage was assessed by imaging methods such as stereo-microscope, electron microscopy with energy-dispersive X-ray, or SEM. It is worth mentioning that all the researchers noticed some differences provoked not only by the conditioning method but also by a chosen material. In most studies, no differences between acid or laser conditioning were noticed, but a combination of laser irradiation and acid etching resulted in lower microleakage [65,67].

The study on twenty bovine incisors concluded that conditioning the surface of enamel provokes a higher bond strength under artificial aging. An ultrasound enamel preparation was compared with classical bur preparation on pit and fissure sealing in the context of caries prevention [68]. Fissures of extracted third molars were prepared in different ways and sealed and assessed with SEM. The study shows that conventional bur preparation prior to sealing gives better retention and may be more effective in caries prevention than ultrasound preparation. Another approach is demonstrated in a study that prepared an enamel not only with etching, but also with combinations with the use of bonding agents or chlorhexidine digluconate [69]. Authors of this 6-month in vitro research claimed that microleakage reduction is most effective in cases of conventional acid etching alone or with a one-bottle adhesive, while it is increased by applying chlorhexidine digluconate. Conventional etching was also compared to the self-etch method in a study conducted on third molars and shear bond strength was checked. This study showed superiority of self-etch preparation for applying a sealer in comparison to etching, adding that results depend also on the chemical composition of the materials and content of 10-methacryloyloxydecyl dihydrogen phosphate [70]. At that point, it is worth mentioning that in general it is claimed that self-etch systems require selective etching by phosphoric acid anyway [71,72,73]. Conditioning the enamel surface by acid etching or no conditioning at all was also studied [74], and it was concluded that etching the surface promotes adhesion of the sealant and enamel.

Another aspect of adhesion considering composite materials is bonding procedures. Usually considering dental sealants, usage of a bonding agent is not recommended by the manufacturer. However, it is regarded to use an adhesive to obtain better retention of composite materials. As mentioned at the beginning of this paragraph, adhesion is obtained by fulfilling the pores in the hard dental tissues by resin monomers that are polymerized and are micromechanically interlocked. Bonding agents penetrate microporosities, which were revealed after etching, by capillary attraction [59]. A clinical trial was performed in a group of children aged 5–15 to compare the retention of sealants with or without using a bonding agent within 12 months. The study concluded that ethanol-based bonding agent significantly increased the retention of sealants on different surfaces of teeth [75].

Taking all the above-mentioned studies into consideration, we may assume that conditioning the enamel prior to sealing has an impact on quality of sealing. That means, in general, there is significantly lower microleakage and better retention of the material. However, there are many studies promoting different approaches than those given by the manufacturers, which require further assessment to be commonly accepted. Bonding the enamel surface prior to applying sealant seems to be a promising approach.

### 3.5. Effectiveness of Sealing and Future Perspectives

The effectiveness of sealing depends on different factors. The anatomy of the enamel surface may be listed as an unmodifiable factor that implicates the level of plaque retention and risk of caries development. Complex topography of pits and fissures makes it challenging to maintain good hygiene. Permanent first molars are the most vulnerable to decay [76]. Convoluted anatomy of the enamel may also contribute to inferior flow of the sealing materials into fissures. Enhanced penetration of the material may lead to decreasing risk of microleakage. A study comparing two different sealing materials (the former with fluoride and the latter without) concluded that there were no significant differences in ability to penetrate fissures between two evaluated materials [77]. However, a material’s flow may depend on its chemical formula and conditions during application, including the temperature, light, or humidity of the operating field. It has been already noted that better retention of the sealing material may be obtained due to isolating the operating field with cotton rolls or a rubber dam [78]. This leaves an excellent proving ground for researchers. It could be beneficial to evaluate the influence of sealer application conditions on microleakage and determine guidelines for clinicians.

The chemical composition of sealing materials may affect their clinical behavior. As mentioned in the previous chapters, the effectiveness of sealants in long-term caries prevention may depend on microleakage. Marginal integrity can be disturbed by increased shrinkage of the materials. As we may see in Table 1, TF1, CS, or USXT have higher values of shrinkage than HF, EW, or TEC. Moreover, the ratio of matrix to fillers may affect the flowability, tribological, and chemical character of dental sealant. Not only may they influence the application process, but also the behavior of the material in the wet environment of the oral cavity, colonized by bacteria.

**Figure 3:**
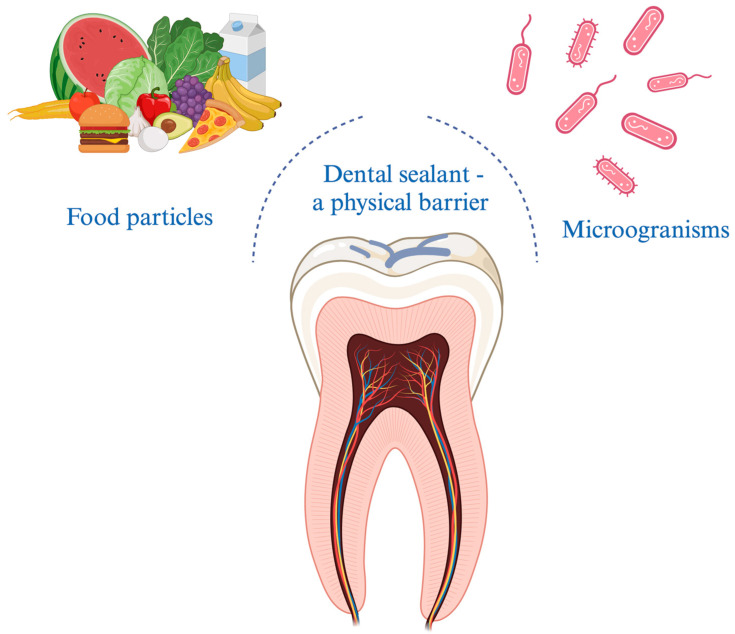
Correctly applied fissure sealant constitutes a barrier against microorganisms and food residues accumulation.

Adhesion, mentioned in previous paragraph, certainly affects marginal tightness of the filling and the appearance of microleakage. As concluded, proper surface preparation may contribute to better adhesion and lead to a clinical success of sealing pits and fissures. Etching the enamel surface is highly recommended by manufacturers. However, there are studies pointing out a superior effect of other surface preparation methods including mechanical preparation [78]. Some authors suggest using pumice or air—polishing instruments [76]. As demonstrated in review with meta-analysis, there are plenty of studies indicating laser preparation as an effective pretreatment method [79]. Figure 3 depicts correctly applied fissure sealant, which allows to decrease microleakage and prevents secondary caries, thus making the sealing procedure more effective.

## 4. Discussion

Caries is a common disease. Children are more prone to caries than adults due to the challenges of maintaining proper hygiene and the greater susceptibility of milk teeth to caries. It is estimated that nowadays, 621 million children in the world suffer from tooth decay. In Europe, it affects 20 to 80% of children, depending on the level of development of the country [80]. Fissure sealants are commonly used materials, especially in the pediatric dentistry. Sealing is an effective way to prevent caries in permanent molar and premolar teeth. Lacquers are also utilized in case of minor carious lesions located in the fissures, as well as the PRR method. The content of fluoride in the materials enables the remineralization of hard tooth tissues and has a bactericidal effect. These are key features for young people who often have poor oral hygiene [81,82,83]. A study of first permanent molars of children from rural areas showed a 44% reduction in risk of dental caries comparing to no sealant use in the 3 years follow-up [84]. 

Numerous studies prove the high effectiveness of fissure sealants in prevention of caries formation. According to Fernandez Barrera M.A., the differences between the effectiveness of individual products of different companies are not clinically significant [85]. Considering materials used in the dental office, their price may be regarded as an important economical factor. While dental caries is a very common health problem globally and dental sealants are perceived as accessible mean of caries prevention, the differences in their prices may be significant. Therefore, it is important to choose the optimal material that will have good tissue retention and effectively prevent caries formation. Multiple examples of research clearly indicate the high effectiveness of fissure sealants in caries prevention [86]. However, the chemical formula and mechanical properties of the material are not the only factors that determine its efficacy. The clinical success of sealing pits and fissures is influenced by the operator’s dependent factors and cooperation with the patient. Furthermore, it is worth noting that sealing materials are compared to another method of prevention—fluoridation. Nonetheless, the application of fluoride usually considers full dentition present in the oral cavity of the patient instead of selected teeth surfaces. It should be emphasized that properly applied sealing does not have to be repeated as regularly as fluoridation [87]. 

The clinical study of Muller-Bolla M et al. regards dental sealants with and without fluoride and their comparison. However, the authors do not pay attention to the differences in their effectiveness, which is noticed by Ivor G Chestnutt in his commentary [88,89]. Instead, the authors pay more attention to the shape of the fissure and the presence of caries. Moreover, the authors do not point out significant differences between fluoride varnish and fissure sealant. Some studies indicate greater effectiveness of varnishes, the application of which is easier and faster.

Although this paper focused on fluoride application and its role in caries prevention, there are other mechanisms affecting processes of remineralization. Amorphous calcium phosphate (ACP) is also added to dental materials in order to promote enamel remineralization and/or inhibit demineralization [90,91,92]. Release of calcium and phosphate ions from a fissure sealant containing ACP and re-release capacity of these ions when charged with a solution containing casein phosphopeptide-amorphous calcium phosphate (CPP-ACP) are mechanisms applied in caries prevention [93]. Some surface pre-reacted glass ionomer sealants recently introduced to the market also show promising effects in the aspect of remineralization [94].

Attention should be also paid to the lack of sufficient evidence to clearly state which of the methods of caries prevention is the most effective [83,95,96]. 

This study has several limitations resulting from the fact that it considers a very broad topic. However, it is mainly focused on the chemical formula of dental sealants, its modifications, and their implications. The aforementioned issue is strictly related to the matter of effectiveness in caries prevention. In addition, the study indicates a potential development direction of biomaterial research in caries prevention. Further experimentation may be focused on sealants modifications, leading to increasing their antimicrobial properties and optimalization of clinical behavior. The role of fluoride is marked as crucial for remineralization processes.

As a matter of fact, the market offers a wide variety of dental materials dedicated for sealing. Multiple studies prove their effectiveness in caries prevention. The beneficial effect of sealing pits and fissures depends both on the sealing procedure and choice of material. Modifying dental sealants striving to increase their cariostatic potential may be an interesting direction of dental materials’ development. The research should not only be a R&D focus, but also a subject of interest of scientists around the world.

## 5. Conclusions

To sum up, fissure sealants are compounds proving effective in preventing caries. Their efficacy increases when the appropriate marginal tightness is maintained. Current research results do not clearly indicate which material is the most effective, but the vast majority fulfil their function provided they are applied correctly. However, choice of dental sealant can be dictated by different conditions, among which patient’s welfare seems to be the most important. Caries risk, aesthetic preferences, intraoral conditions, cooperation with the patient, patient allergies, and operator preferences should be considered while selecting the optimal material for sealing. This article showcases shear bond strength, hardness, and shrinkage for various commercial sealants, all of which meet clinical requirements. Some reports suggest that tooth varnishing is an equally effective method, but easier to apply. Further research is undoubtedly needed to determine which material is best suited for application such as a fissure sealant.

## Data Availability

Not applicable.
